# Supplementary material for: A Bipartite Geminivirus with a Highly Divergent Genomic Organization Identified in Olive Trees May Represent a Novel Evolutionary Direction in the Family Geminiviridae
Source: Viruses. 2021 Oct 9;13(10):2035. doi: 10.3390/v13102035 (PMC8540022; doi:10.3390/v13102035)
Supplement: Supplementary file 1 [file viruses-13-02035-s001.zip › viruses-1395254-supplementary.pdf]

Supplementary files

1 Supplementary Tables

Table S1. Specific pairs of primers designed based on the two different contigs (2727bp and 1286bp) to recover full-length DNAs (DNA-A and DNA-B).

sense	DNA-A		DNA-B	
	2727bp contig	full DNA	1286bp contig	full DNA
5'	GCATCAGCAATGCAAACGGA	CTGTGGGCTAGATCTCTAGGC	AAGCGCGTTGAGGAGATTGA	AAGCGCGTTGAGGAGATTGA
3'	ACCTCGGTCGATACGAGACA	GACAAATCGACACGTAAGCGT	AACGAAGCACGATTCAGAGC	AACGAAGCACGATTCAGAGC
5'		TCCGTTTGCATTGCTGATGC		GCTCTGAATCGTGCTTCGTT
3'		TGTCTCGTATCGACCGAGGT		TCAATCTCCTCAACGCGCTT
5'		TTGTGGATCGAACGGCGAT		TTCTACGTATCTGGACCAGT
3'		CGGATAAAGCGAGCATGGTG		AACGAAGCACGATTCAGAGC
5'		ATACCCAATTGTGCTGGACC		ATCGAACGCTATTGGCTGGT
3'		CTGTGGGCTAGATCTCTAGGC		GCCGTACAAACTTTGACAGTCG
5'		ACCTCGGTCGATACGAGACA		ACCTCTGACGTCATTCTGGT
3'		GGTCCAGCACAATTGGGTAT		TATTGGCTTGCCCCACGG
5'				ACACGGATGTGCATGTCTGAA
3'				ATGACAGTCCTCGAATCAGT
5'				TGTGTTGTGAGTTTTGTTATGTTC
3'				CTCTTCCCGATGAAATCAAACA

Table S2. Genera, species, acronyms, and GenBank accession numbers of the DNA-A (full DNA, Rep, CP) and DNA-B (full DNA, NSP and MP) of the geminiviruses used in this study.

**Genera**	**Species**	**acronym**	GenBank accession numbers

			DNA-A			DNA-B		
			Full DNA	Rep	CP	Full DNA	NSP	MP
Begomovirus	*Euphorbia mosaic virus*	EuMV	KJ647290.1	AID07494.1	KJ647290.1	KJ647291.1	AID07497.1	AID07498.1
(NW)	*Squash mild leaf curl virus*	SMLCV0	AF421552.1	AAL79818.1	AAL79817.1	AF421553.1	AAL79822.1	AAL79821.1
(SLCV clade)	*Squash leaf curl virus*	SLCV	DQ285016.1	ABB76210.1	ABB76209.1	DQ285017.1	ABB76215.1	ABB76214.1
	Pepper golden mosaic virus	PepGMV	AY928512.1	AAY16452.1	AAY16451.1	AY928513.1	AAY16456.1	AAY16455.1
	Melon chlorotic leaf curl virus	MCLCuV	AY064391.1	AAL47673.1	AAL47672.1	-	-	-
Begomovirus	*Abutilon mosaic virus*	AbMV	LN611623.1	CEG02245.1	AGI42760.1	LN611625.1	CEG02250.1	CEG02249.1
(NW)	*Corchorus yellow spot virus*	COYSV0	DQ875868.1	ABI85396.1	ABI85395.1	DQ875869.1	ABI85400.1	ABI85399.1
	Tomato golden mosaic virus	TGMV	JF694490.1	AEI91450.1	AEI91449.1	JF694491.1	AEI91455.1	AEI91454.1
	Bean golden yellow mosaic virus	BGYMV	DQ119824.1	AAZ29484.1	AAZ29483.1	DQ119825.1	AAZ29488.1	AAZ29487.1
	Jacquemontia yellow mosaic virus	JacYMV	KF661331.1	AHX57826.1	ARM20159.1	NC040181.1	YP009547925.1	YP009547924.1
Begomovirus	*Corchorus golden mosaic virus*	CoGMV	DQ641688.1	ABG26007.1	ABG26006.1	DQ641689.1	ABG26114.1	ABG26113.1

(OW)	*Horsegram yellow mosaic virus*	HGYMV0	AJ627904.1	CAF29513.1	CAF29510.1	-	-	-
	South African cassava mosaic virus	SACMV	AF155806.1	AAF34895.1	AAF34893.1	AF155807	AAF34900.2	AAF34899.1
	African cassava mosaic virus	ACMV	FM877473.1	CAT00008.1	CAT00005.1	-	-	-
	Indian cassava mosaic virus	ICMV	AY730035.2	AAU29485.2	AAU29483.1	AY730036.2	AAU29490.2	AAU29489.2
Mastrevirus	*Maize streak virus*	MSV	AF329889.1	AAK73474.1	AAK73472.1	-	-	-
	Chickpea chlorosis Australia virus	CpCAV	NC022131.1	YP008472705.1	YP008472703.1	-	-	-
	Tobacco yellow dwarf virus A	TYDV-A	JN989443.1	AFD63079.1	AFD63080.1	-	-	-
Topocuvirus	*Tomato pseudo-curly top virus*	TPCTV0	X84735.1	CAA59223.1	CAA59221.1	-	-	-
Turncurtovirus	*Turnip curly top virus*	TCTV	GU456685.1	ADJ58423.1	ADJ58421.1	-	-	-
	Turnip leaf roll virus	TLRV00-1	KT388086.1	ALR86871.1	ALR86868.1	-	-	-
	Turnip leaf roll virus	TLRV00-2	KT388088.1	ALR86883.1	ALR86880.1	-	-	-
Curtovirus	*Pepper yellow dwarf virus*	PeYDV	EU921828.1	ACH48060.1	ACH48057.1	-	-	-

	Beet curly top virus	BCTV	AF379637.1	AAK59260.1	AAK59258.1	-	-	-
	Horseradish curly top virus	HrCTV	U49907.1	AAB18926.1	AAB18924.1	-	-	-
	Spinach severe curly top virus	SSCTV	GU734126.2	ADM64623.2	ADM64621.1	-	-	-
Eragrovirus	*Eragrostis curvula streak virus*	ECSV00	FJ665634.1	ACO88029.1	ACO88028.1	-	-	-
Capulavirus	*French bean severe leaf curl virus*	FBSLCV	JX094280.1	AFP53912.1	AFP53911.1	-	-	-
	Euphorbia caput-medusae latent virus	EcmLV	KT214386.1	ANA76366.1	ANA76364.1	-	-	-
	Alfalfa leaf curl virus	ALCV	KT214373.1	ANA76277.1	ANA76275.1	-	-	-
	Plantago lanceolata latent virus	PLLV00	KT214389.1	ANA76386.1	ANA76384.1	-	-	-
Becurtovirus	*Beet curly top Iran virus*	BCTIV	KP410285.1	AKJ80161.1	AKJ80158.1	-	-	-
	Spinach curly top Arizona virus	SCTV00	HQ443515.1	ADV51529.1	ADV51528.1	-	-	-
Grablovirus	*Prunus geminivirus* A	PrGV-A	NC 043533.1	YP009666822.1	YP009666819.1	-	-	-
	Grapevine red blotch virus	GRBV	NC022002.1	YP008400117.1	YP008400114.1	-	-	-
Maldovirus	*Apple geminivirus*	AGV	KM386645.1	AJZ68898.1	AJZ68896.1	-	-	-

	Grapevine geminivirus A	GGV-A	NC 031340.1	YP009305428.1	YP009305425.1	-	-	-
Citlodavirus	*Citrus chlorotic dwarf associated virus*	CCDaV	JQ920490.1	AFN40144.1	AFN40145.1	-	-	-
	Mulberry mosaic dwarf associated virus	MMDAV0	KP303687.1	AJW66421.1	AJW66419.1	-	-	-
	Olea Europaea Geminivirus	OEGV	MW316657	QUS52980.1	QUS52977.1	MW316658	-	QUS52981.1

Table S3. Percentages of homology between OEGV-PT DNA-A (full DNA, Rep, TrAP, REn and CP) and DNA-B (DNA-B and MP) and selected geminivirus (DNAs and predicted proteins).

	DNA-A					DNA-B	
**Species**	full	Rep	TrAP	REn	CP	full	MP
	Percentage of homology (%)						
EuMV	59.8	50.7	36.9	28.1	20.8	59.1	30.1
SMLCV0	60.6	51.6	34.7	23.8	21.8	58.0	29.5
SLCV	62.1	50.9	38.7	25.4	21.3	60.5	29.4
PepGMV	60.7	49.7	38.7	35.2	22.3	57.5	27.9
MCLCuV	59.6	50.0	35.5	25.4	21.4	-	-
AbMV	59.3	48.8	39.5	35.9	22.5	58.1	27.9
COYSV0	59.0	50.3	39.2	32.3	22.5	58.7	29.1
TGMV	60.3	53.0	41.6	34.1	23.2	59.0	30.1
BGYMV	59.7	51.4	36.9	28.1	20.7	58.9	28.6
JacYMV	58.5	52.7	37.0	32.0	20.4	57.1	27.9
CoGMV	57.9	46.2	28.3	29.7	24.4	59.0	28.9
HGYMV0	58.4	47.8	31.0	23.3	22.1	-	-
SACMV	58.1	51.8	28.9	33.3	21.9	57.4	31.0
ACMV	59.0	49.4	33.3	32.3	21.1	-	-
ICMV	60.1	50.3	27.4	32.3	21.7	58.2	34.1
MSV	56.7	33.3	20.9	-	25.0	-	-

CpCAV	59.6	30.7	21.2	-	30.2	-	-
TYDV-A	56.3	31.2	22.4	-	30.1	-	-
TPCTV0	59.0	49.9	25.0	33.1	22.4	-	-
TCTV	59.2	44.1	20.9	31.1	25.1	-	-
TLRV00-1	60.8	46.6	23.2	30.4	23.3	-	-
TLRV00-2	60.0	46.5	23.2	31.8	22.9	-	-
PeYDV	58.7	48.0	23.6	22.8	25.3	-	-
BCTV	60.4	44.4	22.9	25.2	25.3	-	-
HrCTV	59.1	47.1	16.8	-	22.8	-	-
SSCTV	60.7	51.6	21.3	-	28.0	-	-
ECSV00	58.3	45.4	-	-	23.9	-	-
FBSLCV	57.3	27.4	-	-	26.3	-	-
EcmLV	57.3	40.5	-	21.8	21.8	-	-
ALCV	59.0	40.6	-	20.6	22.7	-	-
PLLV00	57.5	37.5	-	23.3	28.1	-	-
BCTIV	58.0	26.3	13.7	-	26.3	-	-
SCTV00	56.9	27.1	16.8	-	25.3	-	-
PrGV-A	58.7	31.4	15.3	21.2	23.7	-	-
GRBV	-	28.8	16.7	17.7	25.0	-	-
AGV	60.7	51.1	29.1	27.8	21.8	-	-
GGV-A	60.6	47.9	29.6	28.7	24.0	-	-

CCDaV	59.1	30.3	-	-	22.1	-	-
MMDAV 0	57.8	27.8	-	-	22.0	-	-

2 Supplementary Figures

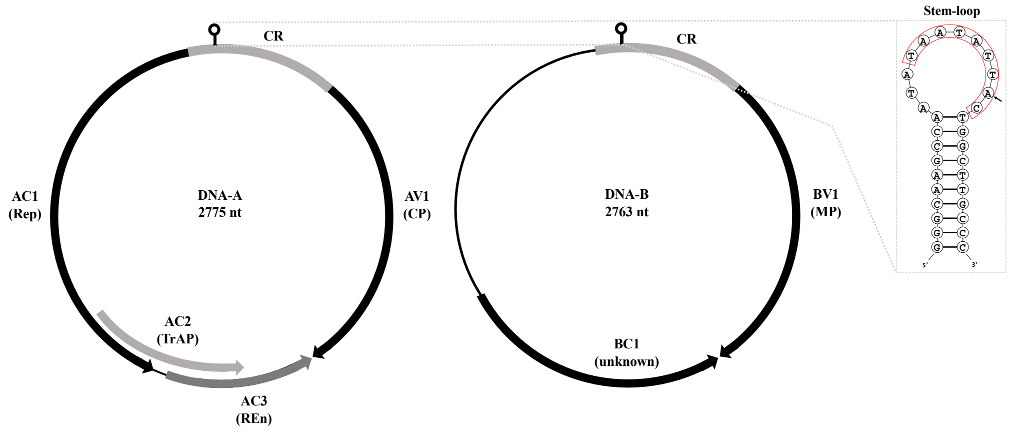

Figure S1. Genome representation of the circular single stranded DNA-A and DNA-B of OEGV-PT. The relative position of four (DNA-A) and two (DNA-B) predicted ORFs. DNA-A: CR, common region sequence; AV1, coat protein (CP); AC1, replication- associated protein (Rep); AC2, transcriptional activator protein (TrAP); AC3, replication enhancer protein (REn); DNA-B: CR, common region sequence; BV1, movement protein (MP); BC1, unknown. Stem-loop of the DNA-A and DNA-B: nonanucleotide sequence "TAATATT↓AC" shown in red arc box (nucleotide position 2769 or 2757 to 2). The ↓ (black arrow) indicates position 1 in the viral genome corresponding to the predicted replication origin of the viral DNA. Arrows denote the virion-sense (V) and complementary-sense (C) geminiviral genes encoded by each component.

Motif I — Motif II

```
OEGV PT ---------------------------MGTPSS--SSSRFCINAKNYFLTYSRCPIS-KEIVLDQIKNFSFP-VNLAFIRVCQEQHS------DGD--NHLHCLLQFSGKYKCRNPRFFD 81
EuMV    -----------------------------MPRN---PNSFRLQARNIFLTYPQCDVP-KDEILQMLQHLPWSVVKPTYIRVAREEHR------DGF--PHLHCLIQLSGKSNIKDARFFD 79
SMLCV0  -----------------------------MPRN---PNSFRFTARNIFLTYPQCDIP-KDEAIQMLEHLQWSVVKPTYIRVAREEHS------DGF--PHLHCLIQLSGKTNIKNARFFD 79
SLCV    -----------------------------MPRN---PNSFRLTARNIFLTYPRCDVP-KEEVLEMLLHLSWSVVKPNYVRVAREEHS------DGS--PHLHCLIQLSGKSNIKDAGFFD 79
PepGMV  -----------------------------MPLP---PKSFRLQCKNIFLTYPQCDIP-KDEALEMLRSLKWSVVKPSYIRVSREEHS------DGF--PHLHCLIQLTGKCNIKDARFFD 79
MCLCuV  -----------------------------MPRN---PKSFRLTTRNIFLTYPKCDVP-KDEVLQMLQGLSWSVVRPTYIRVAREEHS------DGF--PHLHCLVQLSGKSNIKDAGFFD 79
AbMV    -----------------------------MPP----PKKFRIQAKNYFLTYPQCSLT-KEEALSQIQNLETP-VNKKFIKICRELHD------NGE--HHLHVLIQFEGKYQCTNNRFFD 77
COYSV0  -----------------------------MPR----KGSFSIKAKNYFLTYPQCSLT-KEEALSQLQNLNTP-VNKKFIKICRELHE------NGE--PHLHVLIQFEGKYNCTNNRFFD 77
TGMV    -----------------------------MPSH---PKRFQINAKNYFLTYPQCSLS-KEESLSQLQALNTP-INKKFIKICRELHE------DGQ--PHLHVLIQFEGKYCCQNQRFFD 78
BGYMV   -----------------------------MPQ----PQRFRVQSKNYFLTYPRCSIP-KEEALSQLQKIHTA-TNKKFIKVCEERHE------NGE--PHLHALIQFEGKFVCTNERLFD 77
JacYMV  -----------------------------MPR----RGSFSVNAKNYFLTYPQCSLT-KEDVLSQIQNLQTP-TNKKYIKVCRELHE------NGE--PHLHVLIQFEGKYNCTNNRFFD 77
CoGMV   --------------------------------M---SGRFKKQGVSFFLTWPKCPVT-KESALDQIQALTLP-TNIVYIRVCEEKHQ------DGS--PHLHALVQFQKKFICTNCRLFD 75
HGYMV0  ------------------------------MPR---ERRFAINAKNYFLTYPRCPLT-KEDTLEQLLDLSTP-VNKKIHSHLPRAYE------NGE--PHLHVLLQFEGKFHTRNQRFFD 77
SACMV   ------------------------------MPR---AGRFSIKAKNYFLTYPKCTLS-KEAALDQLRQLQTP-TNKLFIKICRELHE------NGE--PHLHALIQFEGKYNCTNQRFFD 77
ACMV    -------------------------------MR---TPRFRVQAKNVFLTYPKCSIP-KEHLLSFIQTLSLP-SNPKFIKICRELHQ------NGE--PHLHALIQFEGKITITNNRLFD 76
ICMV    ------------------------------MSP---PKRFQINAKNYFLTYPRCSLT-KEEALSQIRNFQTP-TNPKFIKICRELHE------NGE--PHLHVLIQFEGKYKCQNQRFFD 77
MSV     -----------------------------MTSS-SSNRQFLHRTANTFLTYPQCPEH-PEIISQRIWDLVGR-WNPLYIICAQEAHE------DGN--MHLHALIQTDKQVRTTDSRFFD 80
CpCAV   -----------------------------MPQTKKPSSSFRLQTKYVFLTYPRCSSD-AESLRDFLWEKLSR-FAIFFIAVATELHQ------DGT--PHLHCLLQLDKRSNIRDPSFFD 81
TYDV-A  -----------------------------MPSAPQKTKSFRLQTKYVFLTYPRCSSS-AENLRDFLWDKLSS-FAIFFIAIATELHQ------DGT--PHLHCLIQLDKRSNIRDPSFFD 81
TPCTV0  -----------------------------MPSN---PKRFQIAAKNYFLTYPNCSLS-KEEALDQLQRLQTP-TNKKYIKVARELHE------NGE--PHLHVLIQFEGKFNCKNQRFFD 78
AGV     ---------------------------MPREPN---TRRFKVQAKNYFLTYPRCSLS-KEEALEQILGLNTP-TNKKFVRVCRELHE------DGE--PHLHVLIQFEGKYTCTNQRFFD 80
GGV-A   MAATSSGRGIYFTPFTPLDGLIELTPIANMPRK---PSSFRLNAKNIFLTYPQCHIS-KESALEQLKAFHYP-IPPVFIKVSAESHQ------DGQ--PHLHALLQFKGKFQTTNQRFFD 10[illegible]
TCTV    -----------------------------MPRT---PRQYRIHAKNIFLTYPHCHLT-KEDALLQLQTIQCP-STKKFIRICRERHD------NGE--PHLHVLIQFEGKIQLYNPRHFD 78
TLRV00  -----------------------------MGRT---NKQYRIQAKNIFLTYPHCTLS-KDEALEQLQNLACP-SNKKFIRISRELHE------NGE--PHLHVLIQFEGKVQICNPRHFD 78
TLRV00  -----------------------------MA-P---PKRFRVAAKNFFLTYPHCFLS-PTEALEQLKQLQIP-VNKKYIRIAKEHHD------DEERTPHLHALLQLEGKFQCTNPRFFD 79
PeYDV   -----------------------------MPR----SPSFQIRAKNVFLTYPRCSII-KEDALEILKNIPCP-SDKLFIRSFQEKHK------DGT--LHLHALIQFKGKAQFRNPRYFD 77
BCTV    -----------------------------MPP----TKRFRIQAKNIFLTYPQCSLS-KEEALEQIQGIQLS-SNKKYIKIARELHE------DGQ--PHLHVLLQLEGKVQITNIRLFD 77
HrCTV   -----------------------------MPRQ---PNSFRIQARNIFLTYPQCTIP-KDEMLQLLQNLNWTVVKPTYIRVAAERHA------DGT--PHLHCLIQLSGKSNIKDCRFFD 79
SSCTV   -----------------------------MPRQ---NKSFRLSSKNIFLTYPQCDIP-KDEVLQLLQNLPWSVVKPTYIRVASERHA------DGT--PHLHCLIQLSGKSNIKDCRFFD 79
ECSV00  -----------------------------MASS---SHRFRIQGRAFFLTYSQCPRE-PKDVGEFLTSHSTLASHVVYVRVQQEKHQ------DGN--NHLHAIVCTSERRDIRDPRIFD 79
FBSLCV  -----------------------------MPRR--NNNSFRLQGKSIFLTYPKCPLT-PLFVIDYLYQLLKN-FNPIYARVCTENHQ------DGE--PHLHCLVQLDKRFNTTSQRYFD 79
EcmLV   -----------------------------MPRQ--TNNTFRLQGKSIFLTYPKCPLL-PIFLIDYLYQLLNN-FNPTYARVCTENHQ------DGE--PHPHCLVQMDKRFNTTNKRFFD 79
ALCV    -----------------------------MPRR---ASNFRLQGKSIFLTYPQCPLI-PMFLIDYLYQLLKN-WDPTYARVCREEHQ------SRE--PHLHCLVQRDKKIDIKDPRFFD 78
PLLV00  -----------------------------MPRQ---PLTYRFQGKSAFLTYPKCDLT-PTSVLDYLYNLLKN-YDIFYARVCQENHQ------DGT--KHLHCLVQLAKRLTTRDPKYFD 78
BCTIV   -------------------MPRAPNRNRPTPNQ---PGYLRFQKKNAFLTYSQIGGDFKDYIFEKLKTLLES-YVILFLAVSLEHHQPTEEQPEGG--FHTHCIIQCDKKLDVNGNLFFN 95
SCTV00  -------------------MARTPSRNRPNQNG---PGYFRFQKKNAFLTYSQIGGDFKDYIFEKLLALLQQ-FEVLFLSVALEHHQPTESNPEGG--FHTHCIFQCNKKLQVNGNLFFN 95
CCDaV   -----------------------------MAST---SSSFRFSAKNIFLTYPKCPCT-KEHLQAFLRLTLAR-FTITYMCVCEELHE------SGD--PHLHAMIQCKKRVETQNPRFFD 78
MMDAV0  -----------------------------MA-S---SSNFRLAAKNVFLTFPQCPES-ITWVMSHLLQILNS-YTVKYACVAEEKHK------DDN--SHFHAIVQLDKKLETRNPNFFD 77
PrGVA   ------------------------------MA----STSFNLRSKNFFLTYPQCPVI-KQFALDFFKSKFT--KTLEYALVSQEKHQ------DGE--PHLHALLIFRKRQCIRNPKHFD 75
GRBV    -----------------------------MAS----SSSFNLRSKNFFLTYPQCPVH-KQFALDFFKSKFQ--KTLDYALIGEEKHQ------NGE--PHLHALLIFKKRQAIRNPKHFD 76
```

GRS motif — Motif III

```
OEGV PT IINPDSDE-----SFHPNIQSARSANDVADYITKG-GSYIDHGQFQEDGR-KRKC--------DRIELDVVYNKALS-ARSKTESLEIIRKNDPRTFYLQHHNISANADRLFPEPIQEYL 185
EuMV    ISHPRRSA-----SFHPNIQGAKDTNAVKNYITKD-GDYCESGQYKVSGG-T------------KSNKDDVYHNAVN-AASAGEALDIIKAGDPKTFIVSYHNVRANIERLFKKAADPWV 179
SMLCV0  LTHPRRAA-----CFHPNIQAAKDTNAVKNYITKE-GDYCESGKYKVSGG-T------------KSSKDDVYHNAVN-AGSAGEALDIIKAGDPKTFLVSYHNLKANIERLFHQPPEPWV 179
SLCV    LTHPRRSA-----RFHPNIQAAKDTNAVKNYITKE-GDYCESGQYKVSGG-S------------KSNKDDVYHNAVN-ACSAGEALDIIKAGDPKTFIVNYHNLLANVERLFQKPPEPWV 179
PepGMV  ITHPRRSA-----QFHPNVQAAKDANAVKNYITKE-GDYCETGHYKVSGV-T------------KANKDDVYYNAVN-AASAVEALDIIKAGDPKTFIVSYHNIKANIERLFTTPPMPWT 179
MCLCuV  LTHPRRSA-----RFHPNIQAAKDTNAVKNYITKE-GDYCESGQYKVSGS-S------------KSNKDDVYHNAVN-AASAGEALDIIKGGDPKTFIVSYHNITANVERLFQKPPEPWV 179
AbMV    LVSPTRSA-----HFHPNIQGAKSSSDVKSYLDKD-GDTAEWGEFQIDGR-SARG--------GQQTANDSYAKALN-AGDVQSALNILKEEQPKDYVLQNHNIRSNLERIFAKAPEPWV 181
COYSV0  LVSPTRSI-----HFHPNIQGAKSSSDVKSYVEKD-GDTIEWGVFQIDGR-SARG--------GQQTSNDAAAEALN-SGTKEEAMKIIKEKLPEKYLFQFHNLSSNLDRIFSKPPKLWE 181
TGMV    LVSPTRSA-----HFHPNIQRAKSSSDVKTYIDKD-GDTLVWGEFQVDGR-SARG--------GCQTSNDAAAEALN-ASSKEEALQIIREKIPEKYLFQFHNLNSNLDRIFDKTPEPWL 182
BGYMV   LVSSTRST-----HFHPNIQGAKSSSDVKAYIDKD-GVTIEWGQFQVDGR-SARG--------GQQSANDSYAKALN-AESIESALTILKEEQPKDYVLHNHNIRSNLERIFVKEPEPWV 181
JacYMV  LVSPNRST-----HFHPNIQGAKSSSDVKSYLDKD-GDVIEWGEFQIDGR-SARG--------GQQTANDTYAKALN-ASTMEESLQIIKEQQPAHYYLQYHNLVANATRIFRKPPEQWI 181
CoGMV   LSHPQNSR-----QFHCHIETARSSSDAKSYIEKD-GVFCEWGTFQVDGR-SARG--------GQQTVNEAYAKALN-SGSKDEALNIIKELVPKDYVLQFHNLNQNLERIFAPPVNVFE 179
HGYMV0  LVSSTRST-----HFHPNIQAAQSCSDVKQYMEKD-GDVLDHGTFQIDGR-SARG--------GKQSANDAYAEALN-CGSKSQALDILKEKAPKDYILQFHNLNCNLNKIFISPIQAYV 181
SACMV   LISPSRST-----HFHPNIQGAKSSSDVKSYLDKD-GDTIQWGEFQIDGR-SARG--------GQQSANDAYAKALN-AASKTEALNVIRELAPKDFVLQFHNLNSNLDRIFQEPPIPYI 181
ACMV    CVHPSCST-----NFHPNIQGAKSSSDVKSYLDKD-GDTVEWGQFQIDGR-SARG--------GQQSANDAYAKALN-SGSKSEALNVIRELVPKDFVLQFHNLNSNLDRIFQEPPAPYV 180
ICMV    LVSPTRSA-----HFHPNIQGAKSSSDVKSYIDKD-GDTLEWGTFQIDGR-SARG--------GQQSANDAYAAALN-SGCKSEALNILRELAPRDYLRDFHHISSNLDRIFTKPPPPYE 181
MSV     ID-----------GFHPNIQSAMSPNKVRDYILKEPLALFERGTFVPRKK-TFLGNSSKGNSEKKPSKDEIMQDIISHATSKPEYLSMVRKSFPYDWATKLQYFEYSANKLFPDIQEEFI 188
CpCAV   FQ-----------GNHPNIQPAKNSEQVLDYISKD-GSVITRGDFRKHKV-------------SPTKSDDRWRTIIQTATTKEDYLEMIKTEFPHEWATKLQWLEYSANKLFPDIEPPYS 176
TYDV-A  LE-----------GNHPNIQPAKNSEQVLEYISKD-GNVITKGEFKKHRV-------------SPSKSDERWRTIIQTATSKEEYLDMIKDEFPHEWATKLQWLEYSANKLFPPQPEIYQ 176
TPCTV0  LVSPTRST-----HFHPNIQGAKSSSDVNSYVDKD-GDTIEWGEFQIDAR-SARG--------GQQTANDECAEALN-RSSKEEALQIIKEKLPKDFLFCYHNLVSNLDRIFTPAPTPFV 182
AGV     LVSPTRSA-----HFHPNVQGAKSASDVKTYIEKG-GEFLDHGVFQIDAR-SARG--------QGQHLAEVYAEALN-APDASASLQIIKEKDPKTFYTQYHNLSANAAKIFMAPPEPFR 184
GGV-A   LVSPSRSA-----HFHPNIQGAKSSSDVKSYIEKD-GDVISWGEFQIDGR-SSRG--------GVQSANDAYAEALN-SGGKDQALQILKEKAPKDYILHYHHLVGNLGRIFKTPPKEYT 211
TCTV    LRDGGSGR-----ICHPNIQGAKSSSDVKSYIEKD-GDYIDWGEFQIDAR-SARG--------GQQTANDACAEALN-SGTAEAALAVIREKLPKDYIFQFHNLKPNLAAIFNPPPVGYV 182
TLRV00  LRHPSASR-----SFHPNIQGAKSSSDVKSYIEKD-GDYLDWGQFQIDGR-SARG--------GQQTVNDASAEALN-AASAEAALAIIREKLPHDFLFKYHNLKPNLAAIFAPPPAVYT 182
TLRV00  LVSPNRSA-----HYHCNIQGAKSSSDVKTYIEKE-GEYSDWGEFQIDGR-SSRG--------GQQSANDAYAAAIN-TGSKQEALRVIKELAPKDYVLQYHNLNANLAAIFAPPPEIYQ 183
PeYDV   ITHPFNST-----TFHPNLQGAKVTSDVKQYIEKD-GDYIDWGTFQVDGR-SARG--------GQQTANDAAAEALN-AGTAAEALQIIREKLPEKFIFQYHNLKPNLEAIFLPPPDIYQ 181
BCTV    LVSPTRSA-----HFHPNIQGAKSSSDVKSYVDKD-GDTIEWGEFQIDGR-SARG--------GQQTANDSYAKALN-ATSLEQALQILKEEQPKDYFLQHHNLLNNAQKIFQRAPDPWT 181
HrCTV   ITHPQRST-----SFHPNVQSAKDTNAVKNYITKE-GDYCESGQYKVSGG-T------------KANKDAVYHNAVN-AGGVREALDIIKAGDPKTFVTQHHNVKANLEHIFAKPPEPWN 179
SSCTV   ITHPRRSA-----NYHPNVQAAKDANAVHNYITKE-GDYCESGQYKVSGS-S------------KANKDDVYHNAVN-SGGVKEALEIIKAGDPKTFVIQHHNVLANLERIFKKPQETWT 179
ECSV00  FG-----------EFHPKIETCRSVSKSLKYIQKEAGSFYEHGTVPCDKRLTGRK--------RKAEQDEWWHQAVN-SGTIEEALQLVKDNEPRTFWLQHHNLVTNARRIWSEVRAEFV 179
FBSLCV  ISDPNRTG-----VYHPNCQVPRRDADVADYIAKGG-QFEERGILRASRR-S-----------PKKSRDSIWTNIINESTSKSEFLGRVQIEQPYVWATQLRNLEYAANSKWPEQPSVYI 181
EcmLV   IKDPNGVA-----TYHPNCQVPRRDADVADYIAKGG-QFDERGILRASRR-S-----------PKKNRDSIWAAILTESTSKSEFLARVQHEQPYVWATQLRNLEYAANSKWPEPITVFE 181
ALCV    IKDPNGVS-----TYHPNTQIPRRDADVADYISKGG-VFEERGLLRASRR-S-----------PKKSRDSIWTTILTESTSKSEFLSRCRTEQPYTYATQLRNLEYMASREWPEPPTVYQ 180
PLLV00  ITTANTITSGDAQPHHPNIQVPRSDAHVADYIAKDG-NFVERGVLKATRR-S-----------PKKSRDTIWTAILQESTSKPDFLARVQSEQPYTWATQLRNLEYAANSRWPEPPVLYE 185
BCTIV   IILPDG-R-----TIHPRIDGLNAPKRAFEYITKEDTSPRTFGELRLGGR-SPNS---------IGNSNVEWRRILDSSNTKEEFFSNIRESCPTDFVLRWPSILSFANYHFRPVVQPYT 199
SCTV00  IILPDG-R-----TIHPRIDGLNAPKRAWEYITKEDQQPRCFGELRLAGR-SPNR---------LGDSNLEWRRILDSSNTKDEFFNNIRESCPTDFVLRWPSILAFANYHFRPVVQPYL 199
CCDaV   LLSVRRER-----SFHPCIESLKSPAASRKYLMKDG-NYVEEGRFNSRAR-SPQ-----------KDQEKLWRDVLLEATDERSFLNLVRELRPSDFVLRWPAISAFARDNYCRLREPFI 180
MMDAV0  LPQEHSSG-----TYHPNVQKCSSPAAVRKYIQKEG-HFIEHGEFNTRGR-SPV-----------ASAEKIFGEILTLATDEESFLALVRERRPQDYVLRWPSITGFARDHYRRRSIPYV 179
PrGVA   ITVSSH-------SYHPNITKPRNVKDVHEYVSKD-GDHIEFGEKPRLHT-------------RERSREVDFGEYLKISNTREEFFNLVKTNYPYQYILNLQRLEYFANQAWPPMPSPHV 174
GRBV    ITISSH-------SYHPNITKPRDVADVLNYVGKE-GDTVEHGTKPTLKS-------------KKQSRELDFGEFLRVANSKDEFLQLVKQHYPYQYVLNLQRLEYFAAQTWPPTPTPHA 175
```

```
                 RBR interaction motif                                                        Walker A  Motif RxL                       Walker B
OEGV PT   SPFDPQSF--VVPQELQYWVTHNIFSP-----------ESRP------------YRTKSLILEGPSRSGKTLWARSLG---PHNYMFGYLDFNSKLYSNNAWYNIIDDVSPSYLQKKHWK 277
EuMV      PPFPLSSFV-NVPEEMQEWADDYFGRG----------AAARPE------------RPISLIVEGDSRTGKTMWARALG---PHNYLSGHLDFNSKVYSNQVEYNVIDDVNPQYLKLKHWK 273
SMLCV0    PPFPLSSFT-LVPEELQEWADDYFNKG----------SAARPY------------RNISIIVEGDSRTGKTMWARALG---RHNYISGHLDFNSRVFSNDAEYNVIDDVAPQYLKLKHWK 273
SLCV      PPFELSSFT-SVPEELQDWADDYFNEC----------SAARP-------------RPTSIIIEGGSRTGKTMWARSLG---PHNYLSGHLDFNSRVFSNDVKYNVIDDVAPHYLKLKHWK 272
PepGMV    PPYPLSSFN-NVPDDMQHWVAEYFGRS----------SAARPD------------RPISIVIEGDSRSGKTMWARALG---PHNYLSGHLDFNAKVYSNDVEYNVIDDITPQYLKLKHWK 273
MCLCuV    PPFELSSFT-SVPEELKDWAGHYFNEC----------SAARPDLIGKSNSVC--NRPISVIVEGDSRTGKTMWARALG---PHNYMSGHLDFNSRVFSNDVKYNVIDDVAPHYLKLTHWK 283
AbMV      PPFPLSSFT-AVPEEMQEWADDYFGSG----------SAARP------------NRPLSLIVEGDSRTGKTMWARALG---PHNYLSGHLDFNGRVYSNEVGYNVIDDVAPHYLKLKHWK 275
COYSV0    PPFPLSSFT-NVPDEMQEWADDYFGRG----------SAARP------------ERPVSLIVEGDSRTGKTMWARALG---PHNYLSGHLDFNARCYSNEVEYNVIDDVAPHYLKLKHWK 275
TGMV      PPFHVSSFT-NVPDEMRQWAENYFGKS----------SAARP------------ERPISIIIEGDSRTGKTMWARSLG---PHNYLSGHLDLNSRVYSNKVEYNVIDDVTPQYLKLKHWK 276
BGYMV     PPFPLSSFI-NVPVVMEEWVDNYFGRG----------SAARP------------ERPISIIIEGDSRTGKTMWARSLG---PHNYLSGHLDFNSRVYSNAVEYNVIDDISPNYLKLKHWK 275
JacYMV    PPFPLSSFN-NVPEVLQEWADNYFGRD----------PAARP------------LRSKSIIIEGDSRCGKTMWARALG---KHNYLAGHLDFNAKCYSDDVDYNVIDDVSPSYLKLKHWK 275
CoGMV     PPFPLSSFN-NVPAVINQWVNDNIMDA-----------AARP------------FRPISIIIEGPSRTGKTLWARSLG---RHNYLCGHLDLSPKVYSNEAWYNVIDDVDPHYL--KHMK 270
HGYMV0    SPYSIDTFS-RVPEYISSWASENVR------------SRCAP------------ERPISIVIEGDSRTGKTMWARALG---PHNYLCGHLDLNDKIFSNEAWYNVIDDVDPHYL--KHFK 271
SACMV     SPFLSSSFT-HVPEELEDWVSENVM-G----------FAARP------------WRPSSIVIEGDSRTGKTMWARSLG---PHNYLCGHLDLSPKVYSNDAWYNVIDDVDPHYL--KHFK 272
ACMV      SPFPCSSFD-QVPDEIEEWVADNVR-D----------SAARP------------WRPNSIVIEGDSRTGKTIWARSLG---PHNYLCGHLDLSPKVFNNDAWYNVIDDVDPHYL--KHFK 271
ICMV      NPFPLSSFD-RVPGELDEWFHENVM-G-----------RARP------------LRPRSIVIEGDSRTGKTMWSRALG---PHNYLCGHLDLSPKVYNNDAWYNVIDDVDPHYL--KHFK 271
MSV       NPHPTSEPDLLCNESIKDWLQPNIYQQ-----------ADNG------------SRKQSLYIVGPTRTGKSTWARSLG---RHNYWQNNVDWSS--YNEDAIYNIVDDIPFKYC--PCWK 278
CpCAV     SPFPNE-FL-QCDEEITEWLNRDLYVV-----------SVES------------YHLLHPNLSIQTATEDLIW-------------------------MDEYTRNPNNSNTEEDPSTSAD 246
TYDV-A    STFTEE-DL-QCHEDLQLWRDQHLYHV-----------SVDA------------YRLIHNVT-LVEATSDLVW-------------------------MDDISRNLEGLEPGSPPSTSAD 245
TPCTV0    PPFQLSSFT-NVPEDMQEWADDYFGVS----------AAARP------------MRYKSIIIEGESRTGKTMWARSLG---PHNYLSGHLDFNSRVYSNSALYNVIDDVTPHYLKLKHWK 276
AGV       CIYLSSSFN-NVPEELDDWVSVNIVSD----------PAARP------------RRPKSIFIEGETRTGKTEWARSLG---PHNYLCGHLDLSPKVFSNDALYNVIDDVDPHYL--KHFK 276
GGV-A     PPFSLDSFN-NVPDELWDWVWESGLGSTAFPSELRENAPAGP------------LRPKSLVLEGDSRTGKTLWARALG---KHNYLSGHLDLNDKVFSLDADYNIIDDVDPHYL--KHFK 313
TCTV      PKYNHTQF--VLTDDILDWLESNFFLE----------ESNSPAGPSKYKVIPSIDRPKSIIIEGPSRTGKTLWARSLG---SHNYITGHLDFSTKVYNDDVSYNVIDDVDPHYLKMKHWK 287
TLRV00    PPFTHTQF--NIPEEIRQWLNNNFILE----------EASSPAGPTKHKRIPSIERPQSIIIEGPSRTGKTLWARSLG---THNYITGHLDFSARVYHDDVEYNVIDDVDPHYLKMKHWK 287
TLRV00    PPYTHTQF--ILPQDIQAWVDTNFTQE----------PDTPPAGPTKHNRIPSIERPQSIIIEGPSRTGKTLWARSLG---THNYITGHLDFSARVYHDEVEYNVIDDVDPHYLKMKHWK 288
PeYDV     PPLSLSSFT-RVPEIIQEWADSYFGVD----------AAARP------------FRYNSIIIEGDSRTGKTMWARSLG---PHNYISGHLDFSLKTYNDDVLYNVIDDVDPNHLKMKHWK 275
BCTV      PLFPLSSFT-NVPEEMQEWADAYFGVD----------AAARP------------LRYNSIIVEGDSRTGKTMWARSLG---AHNYITGHLDFSLRTYYDEVEYNVIDDVDPTYLKMKHWK 275
HrCTV     PPYPLSSFT-NVPEEMQEWADDYFGRP----------VADSH-------------KAKSIIIEGNSRTGKTCWARALG---THNYLCGHLDFNMRVYSNEVEYNVIDDVAPTYLKMKHWK 272
SSCTV     PPFPLSSFN-NVPDEMQEWADNYFGQP----------IESRPI------------RPKSLIIEGDSRTGKTCWARSLG---KHNYLSGHLDFNPRVYSNEVEYNVIDDIAPSYLKLKHWK 273
ECSV00    PKYSENSF--SVPRVLSDWVTNNLRA------------DPLP------------DRPLSLIIEGDSRTGKTAWARSLG---RHNYLSGHLDLNGAVFDNEASYNVIDDVNPKYL--KHWK 268
FBSLCV    PKWTV--FN-NVPEPIREWADTNLFT------------VS--------------LQSMQLV-QPEISVTDMQWAHNLT---E----------D--FITDEWIGNSEDPVVQSF----SG 251
EcmLV     PRFIN--FP-RIPEPIQQWAETNLFT------------DQKP------------DRPITLIIEGPSKTGKTAWARSLG---RHNYYCGGVDFSN--YDVFALYNVIDDIPFQFL--PCKK 267
ALCV      PRWTH--FP-SVPESIKQWADENIFT------------DQKP------------DRPLTLIIEGPSKTGKTAWARSLG---RHNYFCGGVDFSF--WDNHASYNVIDDIPFQFL--PCKK 266
PLLV00    PRYRA--FP-NLPEPIKQWAETNLYT------------DHRP------------DRPITLIIEGPSKTGKTAWARSLG---RHNYYCGSVDWSA--YDKHAMYNVIDDIPFQFL--PCKK 271
BCTIV     PRWTE--FS-RLPDTIKEWAEQNIYFV---------------------------SSDCLHYEICENCRGAILQDRQMT--LEEYFHLDRLSRDLACSEDTNVLNLSRSQTTDQS------ 281
SCTV00    PRWTE--FT-RLPDQIKEWAEHNIYFV---------------------------SSHCLNYELCNNCRGSILQDWEIS--IAEHYHIERLGDELTCSENPNDLNSSRNQTTDQS------ 281
CCDaV     PAFTE--FP-NLPEHVKQWAQQNILCV---------------------------SKPFLQYELCYSCCPKAIFETECPINLQHHFWCDEHR------------NQTPSPT---------- 248
MMDAV0    PRWTD--FP-GLPEPIQQWAKDNILFV---------------------------SRQWADSVLCYRCHDDFWWEVGLT--VPQQYMCNRS--------------PSPSQT---------- 243
PrGVA     RKWST--WN-NLPPEVLQWVNTELFMV-------RKDTARDVF-----------SVAQQNIMHHETYNVKAIIDDMVD---TH-----YIEYMERLYESDNVTDVAGDEPAEEVTSD--- 262
GRBV      NQWTA--WN-NLPPEIHLWVQNELYVV-------RKSSAQRNL-----------GPGINNFLEQETKRVQHIIDDKLD---AY-----YLDYVERIYNTDDELNNAGSDAPKEEEPGD-- 264

                  Motif C
OEGV PT   ELIGVQHNWVSNVKYGKPIQIKG-GIPSIVLCNPGSEEYSYKTFLDKDANSSLKIWTETNAEFFFAESAFYRSEASAMQTEETSSDQA--------------- 364
EuMV      ELIGAQRDWQSNCKYGKPVQIKG-GIPSIVLCNPGE-GGSYKDFLDKEENASLRAWTLKNAKFVFLNSPLYQATAQDCQEESHTSTTD--------------- 359
SMLCV0    ELIGAQRDWQSNCKYGKPVQIKG-GIPSIVLCNPGE-GASYKDFLDKAENTALKAWTLKNAKFIFLESPLYQSTAQDC------------------------- 349
SLCV      ELIGAQRDWQSNCKYGKPVQIKG-GIPSIVLCNPGE-GSSYKDFLDKAENSALREWTEKNAKFIFLEGPLYQSTAQDC------------------------- 348
PepGMV    ELIGSQKDWQSNCKYGKPVQIKG-GIPSIVLCNPGE-GSSYIAFLNKEENASLRAWTLKNAQFVILHAPLYKGTAQDS------------------------- 349
MCLCuV    ELIGAQRDWQSNCKYGKSVQIKG-GIPSIVLCNPGE-GASYKDFLDKAENAALREWTLKNAKFIFLNCPLYQSTTQDR------------------------- 359
AbMV      ELLGAQKDWQSNCKYGKPVQIKG-GIPAIVLCNPGE-GSSYKEYLDKEENTGLRNWTLKNAIFITLTSPLYQEGTQAGQEEGHKAAED--------------- 361
COYSV0    ELLGAQKDWQSNCKYGKPVQIKG-GIPSIVLCNPGE-GASYKDFLDKEENSALRNWTLKNAVFITLTSALYQXSTQASQEEGHQEEAD--------------- 361
TGMV      ELIGAQRDWQTNCKYGKPVQIKG-GIPSIVLCNPGE-GASYKVFLDKEENTPLKNWTFHNAKFVFLNSPLYQSSTQSS------------------------- 352
BGYMV     ELIGAQKDWQSNCKYGKPVQIKG-GIPSIVLCNPGE-GSSYKDFLDKEENRALHNWTIHNAIFVTLTTPLYQSTAQDCQT----------------------- 353
JacYMV    DLIGAQTQWQTNCKYGKPIMIKG-GVPSIVLCNPGE-GSSYKDFLEKEENASLKRWTLYNAEFVFLDSPLYQTSTQGSQEEGSPA------------------ 358
CoGMV     EFMGAQRDWQSNCKYGKPIQING-GIPTIFLCNPGP-TSSYKEFFEEEKNKAINDWAKKNVIYVTIEEPFFNTTNQESTSALEESNSSETN------------ 359
HGYMV0    EFMGAQRDWQSNVKYGKPTHIKG-GIPTIFLCNPGP-KSSYKEYLDEADNASLKVWASKNAEFYTLKEPLFSSVDQSATQGCQEASNSTLSN----------- 361
SACMV     EFMGAQRDWQSNTKYGKPIQIKG-GIPTIFLCNPGP-TSSYKEFLDEEKNQSLKAWALKNATFITLHEPLFSSAHQSPTPHRED------------------- 354
ACMV      EFMGSQRDWQSNTKYGKPVQIKG-GIPTIFLCNPGP-TSSYKEFLDEEKQAALKTWALKNAIFITLTEPLYSGSNQSQPQTIQEASHPT-------------- 358
ICMV      EFMGAQRDWQSNTKYGKPVQIKG-GIPTIFLCNPGP-NSSYKEFLDEEKNSALKAWALKNATFISLEGPLYSGTNQGPTQSC--------------------- 351
MSV       QLVGCQKEFVVNPKYGKKKKVQMKSKPTIILANSDE------DWMKEMTPGQLEYFEANCMIYIMSPGEKWYSPPQLPPTEEV-------------------- 355
CpCAV     QLVPERH--------------------------PGQ-EASEDTITSTEESTSQPMTPTPPST----------------------------------------- 281
TYDV-A    QVVPERQ--------------------------HGP-EASEDTITGMGPSTSLSMMTMRPTTSSTTSPSNSSHSGSN-------------------------- 295
TPCTV0    ELIGAQRDWQSNCKYGKPVQIKG-GIPSIVLCNPGG-DTSFQDFLDKEENEALKDWTLYNAVFIKLTEPLYDGTV---------------------------- 349
AGV       EFCGSQRDWQSNTKYGKPLLVKG-GIPTIFLCNPGP-TSSYKEWLDEEKNSNLKNWALGNAVFVFLSAPLFDPTTSSP------------------------- 352
GGV-A     EFMGAQVGWQSNTKYGKPIHVEK-TMPSIFLCNPGP-NSSYKEFLDEEKNAALKNWALKNAIFVSLQEPLFKPQSPRASEDTAQTSQDRQSS----------- 403
TCTV      HLIGAQKEWQTNLKYGKPRIIKG-GIPSIILCNPGD-GASYQNFLDKPENEALKSWSLQNSVFTTIEEPLFNITSDQEVEDSTPTV----------------- 371
TLRV00    HLIGAQKEWQTNLKYGKPRIIKG-GVPSIILCNPGD-GASYKDFLDKSENEALKSWTIQNSAFATITEPLYDNENIEEAEDDPPTV----------------- 371
TLRV00    HLIGAQKEWQTNLKYGKPRIIKG-GVPSIILCNPGD-GASYKDFLDKPENETLKSWTIQNSAFATITEPLYNNENREEAEDNPPTV----------------- 372
PeYDV     HLIGAQREWQTNLKYGKPRVIKG-GIPSILLCNPGE-GSSYQDFLDKSENEALKSWTLQNSIFAKLSGPLFDKDTETSSQGQSPL------------------ 358
BCTV      HLIGAQKEWQTNLKYGKPRVIKG-GIPCIILCNPGP-ESSYQQFLEKPENEALKSWTLHNSTFCKLQGPLFNNQAAASSQGDSTL------------------ 358
HrCTV     ELIGAQRDWQSNCKYGKPVQIRG-GVPTIILCNPGE-GSSFRDFLDKPENDGLRQWTLYNAIFIFLDTPLFDNGIH--------------------------- 346
SSCTV     ELIGAQHDWQSNCKYGKPVQIKG-GIPSIILCNPGE-GSSFKDFLDKDENESLKRWTLYNAVFISISEPLYNKDD---------------------------- 346
ECSV00    EFLGAQKDWQSNLKYGKPVLVKG-GKPAIVLCNSDQ---SYKSFLDCEENHQLRSWTSKNALFVDIQDPLFRGVSLTLREQTREDDPESPMWASDSDPGDQAV 367
FBSLCV    SETGPPSDSNNCLAQQKQAKQHGPGAWVYIIIFVGV-------------------LILVCGIILLLTPS---------------------------------- 301
EcmLV     ELLGCQKDFTVNEKYRKKTRIKG-GIPTIILCNPDQ------SYRIALANSEMYEWSTHNVLHIEIKDPFY-------------------------------- 331
ALCV      ELLGSQRDFTVNEKYRKKTRVPG-GIPTIVLCNPDQ------SYKTALSSSDMYEWE---------------------------------------------- 316
PLLV00    EILGAQKDFTVNEKYRKKTKVPG-GIPTIVLCNPDQ------SYRAALQLHEMYEWSVCNVLHIEIQQPFY-------------------------------- 335
BCTIV     ----------------------D-QEVSTYADHPEQERPSGQDL----------------------------------------------------------- 302
SCTV00    ----------------------D-PEVSTSAVLQGQERPNGQDR----------------------------------------------------------- 302
CCDaV     ----------------------G-PNPSTSAAQADQAKPPGPEV----------------------------------------------------------- 269
MMDAV0    ----------------------G-PSASTSVDQPDPARRNGPDL----------------------------------------------------------- 264
PrGVA     ------------------------------------------------------------------------------------------------------- 262
GRBV      ------------------------------------------------------------------------------------------------------- 264
```

Figure S2. Multiple alignment of the OEGV-PT Rep protein sequences with most representative geminiviruses including some isolates that had not yet been classified into genera, retrieved from the GenBank database. Gray boxes are highlighting several conserved motifs and blue indicates 100% homology between geminivirus. Motifs; I, II, III, GRS, RBR, walker A, RxL, walker B and motifi C. Virus species and genera are: NW Begomovirus (SLCV clade); EuMV, SMLCV0, SLCV, PepGMV, MCLCuV, NW Begomovirus; AbMV, COYSV0, TGMV, BGYMV, JacYMV, OW Begomovirus; CoGMV, HGYMV0, SACMV, ACMV, ICMV, Mastrevirus; MSV, CpCAV, TYDV-A, Topocuvirus; TPCTV0, Turncurtovirus; TCTV, TLRV00-1, TLRV00-2, Curtovirus; PeYDV, BCTV, HrCTV, SSCTV, Eragrovirus; ECSV00, Capulavirus; FBSLCV, EcmLV, ALCV, PLLV00, Becurtovirus; BCTIV, SCTV00, Grablovirus; PrGV-A, GRBV, Citlodavirus; CCDaV, Maldovirus; AGV, GGV-A, and unassigned MMDaV.
